# Lessons learnt from the multi-centre LAparoscopic Versus Abdominal hysterectomy (LAVA) randomised controlled trial

**DOI:** 10.52054/FVVO.16.1.003

**Published:** 2024-03-28

**Authors:** L Antoun, S Bevan, A Mahmud, L Jones, L Middleton, R Woolley, P Smith, B.Z. Fatemah Sairally, E Saridogan, K Cooper, T.J. Clark

**Affiliations:** Birmingham Women’s and Children’s NHS Foundation Trust, Birmingham, B15 2TG UK and Institute of Metabolism and Systems Research, University of Birmingham, B15 2TT, UK; Institute of Applied Health Research, University of Birmingham, Birmingham, B15 2TT, UK; University Hospitals of North Midlands NHS Foundation Trust, Stoke-on-Trent, ST4 6QG; Institute of Applied Health Research, University of Birmingham, Birmingham, B15 2TT, UK; School of Health and Population Sciences, University of Birmingham, B15 2TT UK; Birmingham Clinical Trial Unit, University of Birmingham, Birmingham, B15 2TT, UK; Birmingham Women’s NHS Foundation Trust, Birmingham, B15 2TG UK; Birmingham Women’s and Children’s NHS Foundation Trust, Mindelsohn Way, Edgbaston, Birmingham, B15 2TG, UK;; University College London Hospital, London NW1 2PG and University College London Elizabeth Garrett Anderson Institute for Women’s Health, London WC1E 6AU; Aberdeen Royal Infirmary, NHS Grampian, Aberdeen, AB25 2ZN and University of Aberdeen AB24 3FX; Birmingham Women’s NHS Foundation Trust, Birmingham, B15 2TG UK and University of Birmingham, B15 2TT UK

**Keywords:** Randomised control trial, recruitment, enrolment, laparoscopic hysterectomy, abdominal hysterectomy, equipoise

## Abstract

**Background:**

The LAparoscopic Versus Abdominal hysterectomy (LAVA) randomised controlled trial comparing laparoscopic hysterectomy (LH) and abdominal hysterectomy (AH) closed prematurely on the grounds of futility. Here we identify the challenges faced and lessons learnt.

**Objectives:**

To explore the views and experiences of clinical/research staff in order to understand how these might act as barriers to trial participation and recruitment.

**Materials and Methods:**

Review of the trial progress and collation of the views and experiences of clinical/research staff on all aspects of the trial. Data were collected from transcribed conversations, email, phone, or video conferencing interactions and analysed descriptively.

**Main outcome measures:**

Site set-up milestones, recruitment rates and reasons provided by clinical/research staff for site’s declining to participate. Opinions, preferences and experiences of clinicians/researchers and challenges to participation and recruitment.

**Results:**

The mean time from initial site contact to opening was 253 days and 68 days to randomise their first participant. 265 patients were screened from 13 sites over 13 months, 154 were eligible, and 75 (59%) were randomised. Of the 53 not randomised, 23 (43%) women preferred LH whilst 6 (11%) preferred AH. The main reasons given for failure to recruit or activate set-up in the 21 sites open or in set-up, were lack of research/ clinical capacity imposed by the COVID-19 pandemic and lack of clinician equipoise.

**Conclusions:**

The main reasons for the LAVA trial failure were lack of equipoise amongst surgeons and the adverse impact of the COVID-19 pandemic on clinical/research services.

**What is new?:**

Surgeons’ preference for laparoscopic hysterectomy is not shared by most patients. Many patients prefer an open hysterectomy to a laparoscopic one.

## Introduction

Hysterectomy is a common operation in gynaecology with almost 40,000 hysterectomies undertaken for benign indications annually in the UK (RCOG, 2018). Laparoscopic ‘keyhole’ techniques, as opposed to conventional open surgery, are being increasingly adopted such that almost 50% of benign hysterectomies are done this way (Madhvani et al., 2019). This paradigm shift has been driven by improved technologies increasing feasibility as well as better surgical training. Greater familiarity with laparoscopic approaches and a focus on enhancing recovery and complying with patient expectations, has promoted same-day hospital discharge after surgery (Antoun et al., 2021; Korsholm et al., 2017) albeit evidence supporting both the feasibility and efficacy of such models of care is scarce.

However, there is some evidence of a higher complication rate with laparoscopic hysterectomy, especially injuries to the urinary tract (Aarts et al., 2015) and costs may be higher as expensive, disposable technologies are used (Antoun et al., 2021; Korsholm et al., 2017; Aarts et al., 2015).

The LAparoscopic Versus Abdominal hysterectomy (LAVA) Randomised Controlled Trial (RCT) was commissioned by the National Institute of Health Research (NIHR) to estimate the clinical and cost-effectiveness of laparoscopic hysterectomy compared to open abdominal hysterectomy for women with a benign gynaecological condition. This was a parallel, open, non-inferiority, multicentre, randomised, controlled, expertise-based surgery trial with an integrated pilot qualitative process and health economic evaluations. Despite the high prevalence of laparoscopic and open hysterectomy, the LAVA trial failed to recruit, culminating in closure on the grounds of futility.

RCTs are considered the gold-standard study design in clinical research but they are difficult to conduct (McCulloch et al., 2002; Cook, 2009). The challenges faced may be greater in surgical trials because of stronger preferences and lack of equipoise in the ‘gatekeeper’ clinicians (Davies et al., 2021; Houghton et al., 2020). Moreover, bias arising from the relative proficiency of both the surgeon and surgical team need to be mitigated. Prolonged waiting times for surgery add further challenges, as this can diminish patients’ willingness to participate (Cook et al., 2015). A comprehensive review across surgical specialities showed that more than 20% of trials were discontinued earlier than anticipated, with poor recruitment being the main reason (Korsholm et al., 2017). Failure of surgical trials results in considerable research and resource waste and leaves important clinical questions unanswered (Treweek et al., 2004; Gul and Ali, 2010; Marcellus, 2004). This has financial and ethical implications (Korsholm et al., 2017; Aarts et al., 2015; Townsend et al., 2015) and the suboptimal situation where patients are offered treatments based on opinion rather than a clear scientific evidence.

We conducted qualitative process evaluation during the 9-month pilot phase of the LAVA trial to explore trial feasibility, acceptability, appropriateness, and processes. The findings of this formal qualitative study will be reported separately (available from the NIHR library) and due to the lack of recruitment, the findings are limited primarily to patients. To gain an understanding of the opinions, preferences and experiences of clinicians following the early closure of the study, we undertook a retrospective descriptive analysis of data collected during the trial. We aimed to identify and assess the challenges encountered and reflect upon the lessons learnt from the failure of the LAVA trial. By sharing our findings, we hope to contribute to the effective implementation and successful delivery of future surgical trials in this field.

## Methods

**Figure 1:**
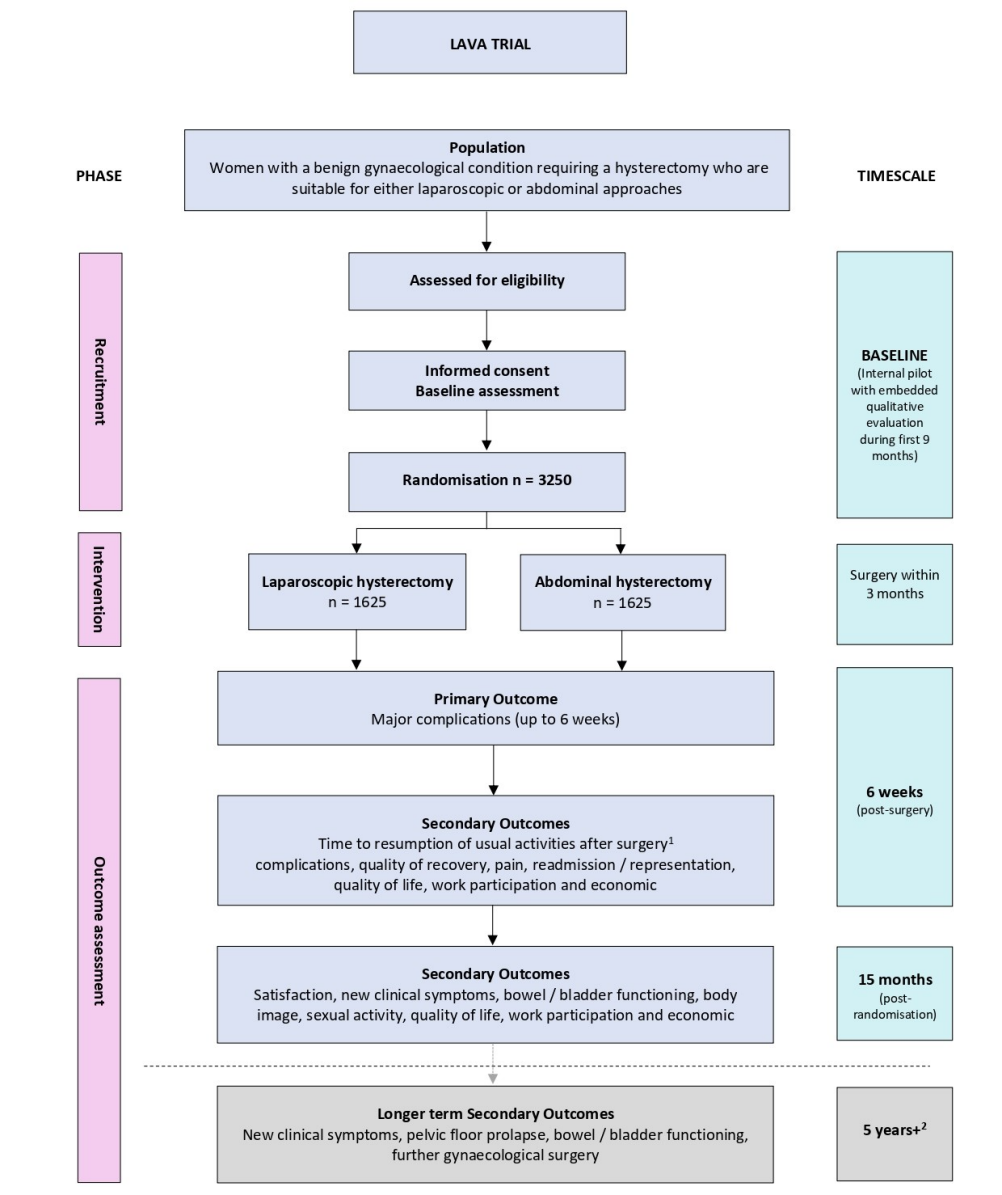
Overview of the LAVA Trial. ^1^ time for surgery to resumption of usual activities will continue to be evaluated until all 8 selected activities have been resumed; ^2^subject to further funding applications.

The LAparoscopic Versus Abdominal hysterectomy (LAVA) trial was designed as a parallel, open, non- inferiority, multicentre, randomised, controlled, expertise-based surgery trial with integrated health economic evaluation and an internal pilot with an embedded qualitative process evaluation. We planned to enrol 3,250 women who required a hysterectomy for a benign gynaecological condition over 36 months from 50 sites. Considering the staggered site set-up and variations in site size and capacity of sites this equated to a mean recruitment rate of 2.5 participants per open site per month over the 36-month recruitment period. Eligible participants would undergo either a laparoscopic or an open abdominal hysterectomy, performed by a surgeon who had self-declared their expertise in either laparoscopic hysterectomy, abdominal hysterectomy or both approaches to hysterectomy. The LAVA trial flowchart is shown in Figure 1. Trial registration: ISRCTN14566195, IRAS ID 287988.

The LAVA trial included a 9-month pilot recruitment period to assess feasibility. Red, amber and green (RAG) criteria were set for the recruitment of sites and participants with actions as follows: If recruitment was =100% of expected, N = 257 (green), proceed to the main trial; if 67% to 99% of expected, N = 172-256 (amber), explore and implement methods to improve recruitment; if <67% of expected, N < 172 (red), and there are no obvious remedial factors, discuss with the Trial Steering Committee (TSC) and consider stopping the trial. The qualitative process evaluation provided dynamic feedback to support decision-making.

The LAVA trial was run by the University of Birmingham Clinical Trials Unit (BCTU). Potential sites were identified by the following methods:

1. Utilising the UK Clinical Research Network (UKCRN) to make all trusts in the UK aware of the LAVA trial
2. Raising awareness of the LAVA trial in editorials of relevant journals (Clark and Saridogan, 2021) as well as speciality conferences and the newsletter of the British Society for Gynaecological Endoscopy (https://www.bsge.org.uk/wp-content/uploads/2022/03/The-Scope-Issue-19.pdf).
3. Identification of potential sites from clinical and co-applicant teams
4. Contacting research active specialists who had previously engaged in clinical research in this field.

The trial management team maintained regular communication with staff at potential sites that had expressed interest in participating in the LAVA trial to ensure a smooth setup of the collaborating sites. This involved addressing any queries they may have had and providing necessary support. Clinical members of the team actively raised awareness of the study and addressed concerns at both individual and group levels. This included speaking at hospital speciality team meetings to disseminate information about the study and address any queries raised.

LAVA trial recruitment commenced when the Birmingham Women’s Hospital (BWH) opened in September 2021. The trial was halted in October 2022 by the NIHR on the grounds of futility due to slower than anticipated recruitment of both sites and participants. We evaluated the progress of the LAVA trial from inception to closure. This descriptive analysis involved a retrospective review of trial activities, including the responsiveness of local Research and Development (R&D) Departments. This included the timelines for site set-up and the rates of participant screening and recruitment. Additionally, we gathered and analysed reasons provided by clinicians and sites for declining their invitation to join the LAVA trial. Collating this information, we aimed to gain insights into factors that influenced the trial’s overall performance. Data are described descriptively, reported as medians for continuous data and as numbers and percentages for categorical data.

We gathered information from clinical/research staff at participating sites, regardless of whether they were in the set-up phase or actively recruiting. These individuals were invited to share their views on the trial protocol and their experiences with patient recruitment. Information was assembled via email, phone, or video conferencing. All sites, including those who had declined participation, were contacted by email at trial closure to provide their views and experiences regarding the challenges encountered in recruiting patients. This inclusive approach aimed to gather valuable insights from stakeholders to better understand the factors that led to the trial’s failure. Dates at which milestones were attained at site set-up were analysed as a proxy of participating trust R&D capacity.

## Results

### Identification and set up of collaborating sites

147 sites (UK NHS Hospitals) were invited to participate in the LAVA trial. Responses were received from 90 sites. Site break down by status at the time of trial closure is shown in (Table Ia). In brief, 31/90 (34%) of responding sites agreed to participate in the LAVA trial, out of which 13/31 (42%) were open for recruitment at the time of trial closure. Five sites (16%) required additional time to set-up due to staff turnover/absences and the remaining 13 were in active set-up (Table Ia). At the time of trial closure, efforts were ongoing to reconnect with 32 sites that initially expressed interest but failed to respond thereafter. Of the 27/90 (30%) sites that declined to participate, 18/27 (67%) provided reasons for their decision. The main reasons cited for declining to participate were a lack of clinician equipoise (i.e. clinician’s preference) in favour of laparoscopic hysterectomy 11/18 (61%), followed by clinician’s uncertainty regarding the trial feasibility 5/18 (28%) and a lack of capacity to undertake the research 2/18 (11%).

**Table Ia:** Sites break down by status at the time the LAVA trial closed.

Status	No/sites
Open	13
Advanced Set Up ^1^	1
In set-up	12
Set up to commence	5
In discussion	6
To follow up	26
No/decline	27
Total	90

Capacity & Capability confirmed / site initiative visit booked or completed.

### Enrolment, recruitment, and randomisation

**Figure 2:**
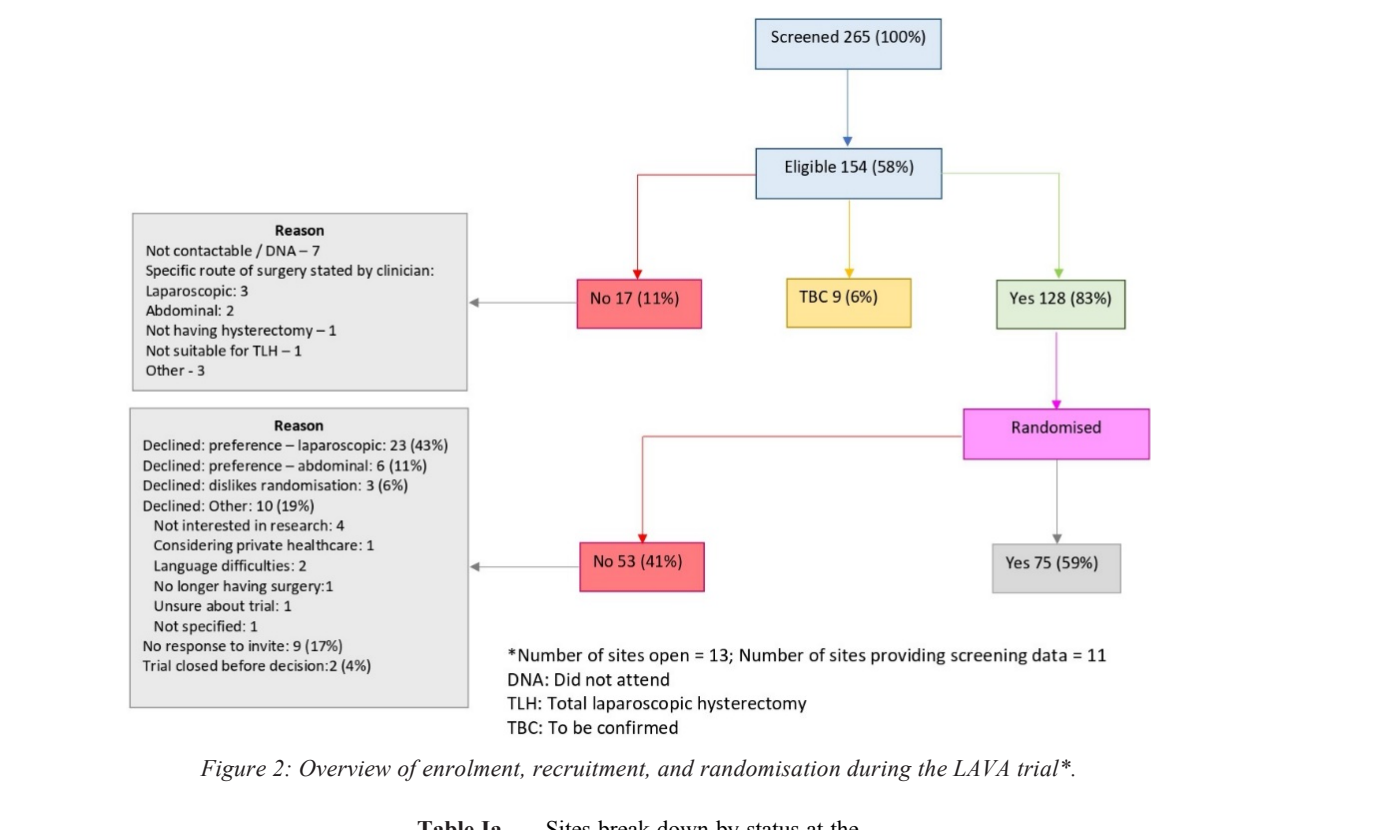
Overview of enrolment, recruitment, and randomisation during the LAVA trial*.

Over 13 months, 265 patients were screened for LAVA, out of which 154/265 (58%) were eligible. Of these 154 potentially eligible patients identified at screening, 128/154 (83%) were invited to participate with 75/128 (59%) being randomised. The main reason for patient non-enrolment was the woman’s preference for the type of hysterectomy as documented in the screening logs; whilst more preferred laparoscopic hysterectomy, several preferred open, abdominal hysterectomy (Figure 2).

The average length of time from initial site contact to receiving the necessary approvals to open was 253 days: median of 232 days and a range of 86 – 449 days. Nine of the 13 open sites (69%) had recruited at least one participant into the study when the LAVA trial was terminated. The mean time between site opening and randomising their first participant was 68 days. Most of the participants were recruited from the lead site (Birmingham Women’s & Children’s Hospital; BWCH) randomising 48 of the 75 (64%) participants and being the only site to exceed the projected monthly recruitment target of 2.5 participants per open site per month. Excluding BWCH, the mean recruitment rate from the other 12 open sites was 0.54 participants per month (Table Ib).

**Table Ib:** LAVA trial recruitment.

	Site	Site open	first recruit	Delay (days)	Sep-21	Oct-21	Nov-21	Dec-21	Jan-22	Feb-22	Mar-22	Apr-22	May-22	Jun-22	Jul-22	Aug-22	Sep-22	Oct-22	Nov-22	Site recruiting for (months)	Total recruited	Mean monthly rate	% Mean monthly rate
1	BWCH	07/09/2021	24/09/2021	17	3	4	3	2	3	3	8	4	3	4	4	0	5	2	0	15	48	3.2	128
2	Royal Bolton	17/01/2022	29/03/2022	71						0	1	0	0	0	0	0	0	1	0	10	2	0.2	8
3	Bradford	19/01/2022	31/05/2022	132							0	0	1	1	1	0	0	0	0	9	3	0.33	13
4	North Durham	07/02/2022	31/03/2022	52							1	1	1	3	0	0	0	0	0	9	6	0.67	27
5	Aneurin Bevan	25/02/2022	11/05/2022	75								0	2	0	1	0	0	1	0	8	4	0.5	20
6	Basildon	10/03/2022	12/05/2022	63									1	1	0	1	0	0	0	7	3	0.43	17
7	Aberdeen	23/05/2022	10/08/2022	79										0	0	0	0	0	0	6	0	0	0
8	Southend	26/05/2022	09/08/2022	75										0	0	2	1	1	0	6	4	0.67	27
9	Portsmouth	08/06/2022	10/08/2022	63											0	1	1	0	0	5	2	0.4	16
10	St Thomas'	09/06/2022	03/08/2022	55											0	1	0	0	0	5	1	0.2	8
11	Lewsiham	17/08/2022	01/11/2022	76													0	0	2	2	2	1	40
12	Manor	08/09/2022																					
13	Macclesfield	29/09/2022																					
				Monthly	3	4	3	2	3	3	10	5	8	9	6	5	7	5	2		75	82	Total recruitment months
				Cumulative monthly	3	7	10	12	15	18	28	33	41	50	56	61	68	73	75			7.6	participants per month
																					0.7	1 participant per open site per month	
			Mean delay	68.91	days from opening to first recruit																		
			Median delay	71	days from opening to first recruit														Recruitment is:		28	% of that predicted	
																						(2.5 participants per open site per month)	

Twenty-one (81%) of the 26 sites open for recruitment or in the set-up process provided either verbal responses that were transcribed and recorded or written responses to our trial closure email enquiring about their opinions and experiences of recruiting to the LAVA trial and why they believed the trial failed (Table II). Lack of research or clinical capacity (resources), reported by 14/21 (67%) of sites, was the main reason given for failure to meet recruitment targets (active sites) or progress in a timely manner to site activation (sites in set up). The other main reason given was lack of surgeon equipoise 11/21 (52%).

**Table II:** Reasons given by sites for failing to meet recruitment targets.

Reasons^1^ (21 sites)		Frequency (%)
Capacity		14 (67%)
	1. R&D departments/research nurses	9
	2. Clinical staff	2
	3. Theatre (prioritisation of cases)	3
Clinical preference to perform laparoscopic hysterectomy (lack of equipoise)		11 (52%)
Impact of COVID pandemic		3 (14%)
Delay in set up		2 (10%)
Confusion over trial protocol		1 (5%)
Language barrier to fill in patient’s questionnaires		1 (5%)

^1^ Reasons were not mutually exclusive.

### The impact of the COVID-19 pandemic

The COVID-19 pandemic had a significant impact on the NHS. This has had a lasting impact both upon provision of patient care and research. Research and Development (R&D) departments have faced challenges in restoring normal capacity due to staff redeployment to cover sickness absences and vacant posts. The pandemic resulted in reduced research and clinical capacity at sites making it difficult for them to participate in research. Prolonged surgical waiting times discouraged surgical research as this would further exacerbate the delay to operate. These factors acted as barriers to trial activation. Three (11%) out of 27 of the declining sites had initially shown enthusiasm to take part but then decided to withdraw their interest due to capacity issues imposed by the COVID-19 pandemic.

Trial recruitment began in September 2021. However, enrolment and recruitment in all sites was paused for three months (December 2021 – February 2022) during the pilot phase because of staffing shortages during COVID-19 pandemic because of sickness and redeployment of research staff to clinical roles (December 2021 – February 2022).

## Discussion

### Main findings

The LAVA trial was halted by the funding body after the pilot phase on the grounds of futility due to poor recruitment of both sites and participants. Thus, with the failure of the LAVA trial it appears that the current trend to fewer abdominal hysterectomies will continue without robustly answering important clinical questions around patient safety, recovery, and cost-effectiveness. Our retrospective descriptive analysis of the progress of the trial, incorporating the views of clinical and research personnel, identified the key reasons for the failure to recruit to the LAVA trial. These reasons are discussed below.

### Lack of interest from the clinical community

The LAVA trial was ambitious for a surgical trial because of its large sample size. However, considering over 34,000 hysterectomies are performed annually for benign disease (excluding prolapse) in the United Kingdom1,2 we believed that the trial was feasible. Furthermore, a large trial was required to robustly answer the most important questions surrounding safety and recovery. We projected that recruitment over 36 months to reach 3250 participants would require 50 sites. However, of the 90 sites responding to our initial expression of interest, only one-third agreed to take part. Whilst this number fell short of our target of 50, this was only the first wave of site recruitment and most likely reflected the lack of capacity issues arising from the COVID-19 pandemic. As our projections factored in a staggered start-up of sites across the whole recruitment period, we were initially encouraged by this positive response rate and believed that more sites would respond affirmatively as the NHS recovered from the pandemic. However, the trial was closed before this assumption could be validated.

### Lack of surgeon equipoise

60% of sites declined the invitation to participate in LAVA citing a lack of surgeon equipoise. Despite the available evidence base not clearly demonstrating the superiority of laparoscopy over abdominal routes, a clear preference for laparoscopic hysterectomy over other approaches was expressed by respondents (ACOG, 2009). The LAVA trial was designed pragmatically without strict eligibility, freeing surgeons to decide which cases they were prepared to randomise. We also designed the trial with an ‘expertise design’ to minimise surgical proficiency bias. It was hoped that surgeons who were not prepared to randomise would still participate as ‘expert surgeons’ for those women randomised by colleagues with equipoise. However, this did not materialise during the pilot phase of the trial for varied reasons that were broadly as follows:

1. The proposed PI or most influential surgeons within a department were self- declared expert laparoscopic surgeons (according to the expert criteria in the LAVA trial protocol) (Clark et al., 2021).
2. Several units pooled their surgical waiting lists and allocated cases deemed suitable for laparoscopic hysterectomy to the laparoscopic surgeons such that abdominal hysterectomies were spread more thinly across the departments and restricted to those with larger uteri.
3. The younger generation of gynaecological surgeons lacked competence or confidence in abdominal surgery because of limited exposure to open, abdominal hysterectomy during their surgical training.
4. The recommendation to aim for same day hospital discharge following laparoscopic procedures (GIRFT, 2020), avoiding prolonged hospitalisation brought even more to the fore with the strain on hospital’s resources imposed by the COVID-19 pandemic.

It is telling that while most sites declining to participate in LAVA cited lack of surgeon equipoise and 50% who were open or in set-up stated this as the reason for poor recruitment. However, the data shows that this certainty was not shared by the very patients the trial was trying to inform. Only 23/154 (15%) eligible patients expressed a preference for LH. Surgeons, by the nature of their job, need to be decisive but they should reflect upon their duty to patients where the evidence base is lacking and the Hippocratic oath of “first doing no harm”. Randomisation, if 1:1, protects 50% of participants from unwanted outcomes, both known and unknown.

### Scepticism over feasibility

The large sample size compounded by the arrival of a major pandemic that strained health services, surgical waiting lists and overall clinical capacity appeared to influence some clinicians’ views on the trial viability. R&D departments were particularly impacted, with major staffing issues due to long-term sickness, unfilled posts, and reallocation of scant resources to other research priorities (e.g. COVID-related trials). Following the restart of non-COVID studies, the LAVA trial was designated new trials like LAVA were the lowest priority for R&D departments who concentrated preferentially on restarting trials that had been suspended during the covid pandemic or new covid related studies.

### Erroneous perception of lack of patient equipoise

Clinicians who declined participation in the LAVA trial, as well as some within participating units, expressed concerns about the feasibility of recruitment, presuming that patients would have a strong preference for laparoscopic hysterectomy as a ‘modern technique’. This perception assumed that patients would prioritise smaller abdominal scars and anticipate quicker recovery with the laparoscopic technique. However, our recruitment data challenged this belief, presenting a more balanced picture. Nearly 60% of eligible women agreed to be randomised, indicating a willingness to consider different types of hysterectomy. It is worth noting that when a preference was expressed that precluded participation nearly a quarter of women preferred open, abdominal surgery.

### Delays in site set-up and lack of R&D efficiency

Only 13 (40%) of the initial 31 sites agreeing to participate in the LAVA trial were open for recruitment when the study was closed. This fell way short of our target of 28 sites by the end of the pilot phase. The trial team maintained regular contact with sites with additional support, when necessary, by the Chief Investigator (CI) through phone and email contact and attendance remotely at departmental meetings to present the LAVA trial and address questions and queries. At many sites the local principal investigators (PI) were keen to move the set-up process along but had little influence over this. The limiting factors were the decisions of local clinical management around clinical priorities and capacity and those of the R&D departments around their priorities, capacity, and efficiency. Sites in the set-up phase would frequently ask for more time or to be re- contacted after a few months to facilitate the evaluation of the changing clinical and research situation. Thus, while many clinicians were keen to get involved with the LAVA trial, they were unable to do so in a timely way.

### Poor recruitment due to capacity and equipoise issues in collaborating centres

Ten of the 13 open sites managed to recruit. However, there appeared to be a noticeable delay in getting the trial embedded, with the median time from site opening to the first patient being randomised was 67 days. The required recruitment rate from trial inception was 2.5 patients per open centre per month. By the time of trial closure, we had achieved an average rate of 0.8 per open centre per month. The lead site (Chief Investigator’s site, the Birmingham Women’s & Children’s Hospital; BWCH) recruited the majority of patients 48/75 (64%) at a rate of 3.2 participants per month, However, it was clear that collaborating sites were struggling to recruit with a mean rate of only 0.44 patients per centre per month.

The experience from the lead site demonstrated that recruitment at expected rates was feasible but this could not be replicated in other centres. While this discrepancy could be anticipated, given the lead site’s responsibility and accountability for the trial, the expectation was that additional high-performing sites would emerge. The fact that this did not occur may reflect the small number of sites open for a reasonable period of time (e.g., six months) before trial termination. It may be because the trial never got a chance to be truly embedded in these sites due to the disruption of the pandemic. The only difference in resources at the lead site was the appointment of a funded research fellow whose role was primarily to engage with actual or potential collaborating sites. Pathways were put in place at the lead site to efficiently identify and introduce the LAVA trial to potential participants by the direct care team after which they were directed to members of the local research team.

Lack of clinical and research capacity was one of the main reasons given by almost 70% of active sites and those in set up, when asked about their experiences of recruiting to the LAVA trial and why they believed the trial failed. The other prevalent reasons given by half these sites related to the preferences of clinicians within sites and a perceived lack of equipoise when potentially eligible patients were identified but were not subsequently invited or randomised.

### Impact of the COVID-19 pandemic

The unpredicted COVID-19 pandemic had a major impact on global health services. The clinical and research infrastructure was put under great stress. Non-essential clinical services were shut down and surgical waiting times rose exponentially, especially those in women’s health where many operations were designated as ‘non-urgent’ (Memon et al., 2020) Consequently, the absence of paid or protected time for clinical PIs made trial engagement difficult whilst restoration and recovery of clinical services took priority. Moreover, R&D departments had to reallocate research priorities and deal with staff shortages because of redeployment of staff to clinical areas and long-term sickness. At least 10% of sites willing to take part in the LAVA trial were lost due to this lack of capacity not to mention those sites that had not responded to the initial invitations of expressions of interest.

All trials need to gain a critical mass of collaborating centres actively recruiting. We encountered difficulties gaining momentum. Once we had finally got five sites up and running there was a second wave of a COVID-19 and this impact upon human resources meant that many R&D staff were redeployed to support clinical services; this intervention also affected research fellows and research nurses. Thus, once the clinical impact of this second wave had peaked, the need to re-engage with open centres and those in set up had to begin again.

### Relation to previous research

The LAVA trial’s sample size of 3250 women would have eclipsed the 25 previous RCTs whose total participant number was 2983 patients. Importantly, it would have addressed the previous criticisms relating to lack of power, expertise bias and invalid outcome assessment especially relating to categorisation of complications and assessing recovery (Chien et al., 2005; Donnez et al., 2004). The LAVA trial was commissioned by the NIHR HTA programme with substantial public funding to help build the necessary evidence base to best inform surgical treatment offered to women undergoing a hysterectomy for a benign condition. The failure of this important surgical trial is regrettable considering the paucity of surgical RCTs (Pollock, 1993).

Recruitment to clinical trials is inherently difficult, yet surgical trials present specific challenges including clinical capacity and interest, mitigating for surgical proficiency/expertise bias, factoring in a delay between consent and/or randomisation and surgery taking place and addressing surgeon equipoise/preference, and surgeons tending to ‘like’ doing things a certain way (Davies et al., 2021). Many of these challenges became even more pronounced during and after the COVID-19 pandemic (Meyer et al., 2021).

The most common patient-reported reasons for non-entry into surgical RCTs are treatment preference or dislike of randomisation (Abraham et al., 2006; Howes et al., 1997), and where treatments are markedly different, there is an increased likelihood of patients or clinicians declaring a preference (Cook, 2009; Stirrat et al., 1992; Kuppermann et al., 2004). It should be noted however, that in the LAVA trial the surgical treatment was the same (hysterectomy) and only the technique differed. This seemed to matter less to patients than to participating surgeons. In addition, recruiting clinicians often struggle to explain concepts such as randomisation and equipoise (Donovan et al., 2014; Wade et al., 2009; Fisher et al., 2012). To try and overcome the latter issue we produced a short video presentation setting out how this was done at the lead site.

The Trial Forge initiative (Treweek et al., 2015) aimed to increase the evidence base for trial decision-making and to improve trial efficiency by providing strategies that could be used across RCTs. A review of 73 multicentre RCTs summarising interventions in various clinical areas found that only 55% recruited their target sample and 45% required an extension of their recruitment time (Sully et al., 2013). Another review of 114 multicentre RCTs investigating assorted interventions including surgical procedures found that fewer than one-third successfully recruited their target sample size within the planned timeframe (Campbell et al., 2007). Therefore, the failure of the LAVA trial is not unexpected but there are lessons that can be learned for future trials to succeed and to avoid research waste.

### Lessons learnt and future research

We present ten “lessons learnt” from the failure of the LAVA trial that could help others planning to conduct their own multicentre surgical RCTs. These lessons are categorised into six themes based on the challenges identified during the trial and as part of this retrospective descriptive analysis (Table III). We hope that these lessons will provide guidance to improve trial efficiency allowing future trials to meet their objectives.

**Table III:** Challenges faced and 10 lessons learnt from the LAVA trial.

Challenges	Lessons learnt
Lack of interest from the clinical community	1. Chase up non-response to initial expressions of interest (EOI) and review the trial feasibility where EOI responses fall short of those expected in the absence of any clear explanation, especially for large trials where many centres needed.
Lack of surgeon equipoise	2. Establish if the proposed treatment is acceptable to all Principal Investigators (PIs) using qualitative research. PI to establish that the surgical team encompasses a critical mass of surgeons prepared to randomise and who have the relevant expertise to undertake the procedures being compared especially in a rapidly evolving surgical field.
Scepticism over feasibility	3. Ensure simplicity in trial procedures and maintain regular engagement with PIs and R&D departments, especially in active sites to troubleshoot local issues, answer delaying queries and promote the trial. 4. Proactively analyse and make changes to the existing study protocol to help set-up and recruitment to mitigate the impact of unanticipated, hostile emerging circumstances.
Erroneous perception of lack of patient equipoise	5. Inform and educate surgeons to prevent misconceptions about patient preferences and willingness to participate in the trial using embedded qualitative research and real-time quantitative data from active sites.
Delays in site set-up	6. Early discussions about site requirements using a site feasibility questionnaire. Prepare and anticipate site needs. 7. Make local Research & Development (R&D) departments more accountable for timely site set up and trial participation even in a challenging clinical environment.
Poor recruitment	8. Communicate regularly with participating sites and use clinicians already recruiting and participants already recruited into the study to advocate for the trial. Continue to express appreciation to participating sites. 9. PI and local R&D departments to confirm site capacity (clinical and research infrastructure) and that clinical colleagues are prepared to take part.
Impact of the Covid-19 pandemic	10. Early review of ongoing trial feasibility, evaluating the speed of restoration and recovery of clinical and research services that have undergone major disruption.

### Strength and limitations

Our overarching, retrospective descriptive analysis takes into account the quantitative and qualitative data from interactions with clinicians and researchers obtained from several sources including telephone, email, teleconferences, site visits, and consultant/research meetings. These data were contemporaneously recorded but not formally collected in a systematic way to enable evaluation via thematic analysis. However, by presenting all data from a range of sources we believe that we have been able to make meaningful and valid inferences. Our data provides invaluable perspectives from clinical and research staff at participating or declining sites that will be valuable to future trials hoping to execute surgical RCTs. Moreover, our qualitative study interviewing patients from recruiting sites will be published separately.

## Conclusion

Large scale, multicentre surgical trials that can answer clinically important questions are rare mainly because they are difficult to execute. This paper summarises barriers faced by a well- designed, multi-centre, substantial trial comparing surgical techniques that was closed due to futility. The challenges encountered and reasons identified that collectively resulted in the failure to recruit both patients and sites have been summarised. Whilst the unprecedented challenge presented by the COVID-19 pandemic could not have been anticipated, most reasons leading to the trial’s failure are identifiable and could potentially be addressed through effective strategies and early intervention.

Overcoming these challenges will require research to identify interventions that can enhance recruitment and targeted investment to support innovative solutions. However, trials need to be made simpler and the bureaucratic process needs streamlining, R&D departments need investment but also to be made more accountable for their performance, especially when trials fail. More radically, to overcome the issue of surgeon prejudice/preference, we should consider only introducing new procedures, or even conventional procedures performed in a different way (e.g. using different approaches, technologies, robotics etc.) into routine practice after formal evaluation. This should comprise adequately powered independently funded trials, as would be the case with the introduction of a new pharmaceutical agent.

We hope that the analysis for the failure of the LAVA trial and the lessons learnt may be transferrable to improve recruitment and successful delivery of future surgical RCTs.
